# Synergistic Anti‐Aging Effects of Adipose‐Derived Stem Cell Extracellular Vesicles Loaded With Natural Compounds

**DOI:** 10.1111/jocd.70021

**Published:** 2025-02-09

**Authors:** Nhan Vo, Diem My Vu, Nam H. B. Tran, Diem D. N. Nguyen, Phuc M. Phung, Hoai‐Nghia Nguyen, Lan N. Tu

**Affiliations:** ^1^ Medical Genetics Institute Ho Chi Minh Vietnam; ^2^ Center for Molecular Biomedicine University of Medicine and Pharmacy at Ho Chi Minh City Ho Chi Minh Vietnam

**Keywords:** exosomes, extracellular vesicles, human adipose‐derived stem cells, nanocosmetics, skin anti‐aging, UV protection

## Abstract

**Background:**

Small extracellular vesicles from adipose‐derived stem cells (ASC‐sEVs) are gaining attentions rapidly for inherent therapeutic values in skin care and cosmetics. However, the optimal combinations of ASC‐sEVs and certain natural compounds for synergistic anti‐aging effects have not been systematically studied.

**Methods:**

Human ASC‐sEVs were purified from culture supernatant of ASCs and multi‐omics datasets of miRNAs, proteins and lipids of ASC‐sEVs were analyzed for pathways regulating skin homeostasis. ASC‐sEVs were then loaded with nicotinamide riboside (NR), resveratrol (RES), vitamin C (VITC), retinol (RET) and arbutin (ARB) at different concentrations by the sonication‐incubation method. Their anti‐oxidant, anti‐wrinkle and anti‐melanogenic effects were tested in vitro using the human keratinocyte HaCaT cells exposed to UVB radiation and human melanocyte B16F10 cells.

**Results:**

Multi‐omics data analysis of ASC‐sEVs identified key bioactive molecules regulating collagen formation, pigmentation, oxidative stress and inflammation. In the in vitro screenings for anti‐aging effects, the compound‐loaded ASC‐sEVs outperformed the sEV‐ and compound‐only treatments. Specifically in UVB‐exposed HaCaT cells, 2 μg/mL sEVs loaded with 20 μg/mL NR, 2 μg/mL RES, 5 μg/mL VITC reduced reactive oxygen species level by 22.0%; while combination of sEVs and 2 μg/mL RES, 2.8 μg/mL RET significantly reduced *MMP3* and upregulated *PLOD1* expressions. B16F10 cells incubated with 2 μg/mL sEVs loaded with 2 μg/mL RES, 0.5 mM ARB had intracellular and extracellular melanin content lowered by 21.4% and 22.4% respectively. All the combinations caused no cytotoxicity.

**Conclusion:**

Our study demonstrated the superiority of ASC‐sEVs to deliver both endogenous biocargos and exogenous compounds to achieve synergistic skin anti‐aging effects.

## 1 Introduction

As the body's largest organ, the skin acts as a protective barrier against pathogens and injuries and is essential for maintaining homeostasis [1]. The natural process of skin aging involves a gradual decline in skin elasticity and firmness, along with the development of wrinkles and age spots [2]. This process can be hastened by factors such as sun exposure, pollution, smoking, and inadequate skincare practices. These elements contribute to oxidative stress, inflammation, and the degradation of collagen and elastin fibers in the skin [3].

Small extracellular vesicles (sEVs), a heterogeneous group of lipid‐bound nanoparticles secreted by cells with a diameter smaller than 200 nm, has drawn much attention in skin therapeutics and aesthetics [4]. Depending on cell sources, isolated sEVs display distinctive properties as they are filled with various proteins, lipids, and nucleic acids such as miRNAs derived from their parent cells. A large body of studies has demonstrated that sEVs derived from human adipose mesenchymal stem cells (ASC‐sEVs) contain a wide range of biological molecules that regulate essential cellular processes in skin health, skin photoaging and dermatological diseases [5]. However, considering the fact that the endogenous cargo of ASC‐sEVs is a complex biological mixture, in‐depth characterization of its composition and specific skin‐related functions is still necessary.

Unlike sEVs, natural compounds have been utilized for centuries for skin care purposes as they are safe, widely accessible, and present a variety of biological effects including anti‐oxidant, anti‐inflammatory, or anti‐pigmentation activities. Their effectiveness, however, hinges on their formulation, as many compounds have difficulty in penetrating the stratum corneum due to the lack of chemical stability and an appropriate solubility coefficient. These include nicotinamide riboside (NR), resveratrol (RES), vitamin C (VITC), retinol (RET) and arbutin (ARB) [6, 7, 8]. To address skin penetration challenges, ASC‐sEVs may serve as a superior nanocarrier, as they can penetrate deeper into the skin and efficiently deliver both their endogenous bioactive cargos as well as exogenously‐loaded compounds. We previously reported a combination of human ASC‐sEVs loaded with NR and RES that provided synergistic effects in both photoaging protection and skin rejuvenation [9].

In this study, we performed bioinformatics analysis of multi‐omic datasets of human ASC‐sEVs to identify key molecules regulating skin aging and homeostasis. We then conducted a systematic in vitro screening to identify the optimal combinations of sEVs and natural compounds to deliver the synergistic skin anti‐aging effects.

## 2 Methods

### 2.1 Human ASC Culture and sEV Production

Human adipose‐derived stem cells (ASCs) (Thermo Fisher, USA) were cultured in MesenPRO RS Medium (Thermo Fisher, USA) at 37°C with 5% CO_2_ as described previously [9]. From passage 5, the cells were switched to an optimized expansion medium consisting of DMEM with 10% FBS and StemMACS MSC Expansion Medium (Miltenyi Biotec, Germany) at the volume ratio of 75%:25%. For sEV collection, the ASCs were seeded at 30% confluency, washed with PBS to remove FBS and then cultured in a harvesting medium consisting of serum‐free DMEM and StemMACS MSC medium at the volume ratio of 50%:50% for 72 h.

### 2.2 sEV Isolation

The sEV isolation and purification protocol was established and described previously [9, 10]. Briefly, the conditioned medium was centrifuged at 300× *g* for 5 min, then at 3000× *g* for 30 min at 4°C and filtered through a 0.22‐μm PES membrane (Merck, Germany). The medium was next concentrated using tangential flow filtration (TFF), followed by two rounds of diafiltration with PBS and then ultracentrifugation using 30% sucrose cushion (Sigma‐Aldrich, Germany) at 120 000× *g* overnight at 4°C. The bottom sucrose fraction was collected and diluted with PBS and further concentrated by ultracentrifugation at 120 000× *g* for 3 h at 4°C using a fixed‐angle rotor (Beckman Coulter, USA). The resulting sEV pellets were resuspended in PBS, aliquoted, and stored at −80°C.

### 2.3 sEV Multi‐Omics Data Analysis

Datasets of miRNA sequencing, proteomics and lipidomics of human ASC‐sEVs were generated in our previous study [9]. Target genes for each miRNA were predicted using the miRTargetLink 2.0 [11]. Pathway enrichment analysis for all miRNA‐targeted genes as well as all proteins in the proteomics data was performed using the Reactome database [12] with a significant false discovery rate set below 0.05. Skin‐related pathways were selected and visualized based on their involvement in the skin structural stabilization, moisture retention, collagen formation, pigmentation and anti‐inflammatory responses. For lipidomics data, all lipid species were categorized into five major classes: sphingolipids (SP), fatty acids (FA), glycerophospholipids (GP), glycerolipids (GL), and sterol lipids (ST). Their respective abundance was calculated and skin‐related biological effects were manually curated from literature.

### 2.4 sEV Loading and Characterization

For each compound, the range of concentrations tested was curated from the literature for skin anti‐aging effects; and the maximal concentration was determined safe by the cell viability assay as described below. Purified sEVs (10^8^–10^9^ particles) were then mixed with compound(s) in 100 μL DMEM, sonicated for 1 h at 35 kHz, 35°C–45°C using the Elmasonic Select 60 (Elma, Switzerland), and then incubated for 4 h at 25°C with continuous shaking at 600 rpm. The loaded sEVs were washed twice with PBS using the 30 kDa MWCO Pierce protein concentrators (ThermoFisher, USA). Compounds used in the study included nicotinamide riboside (NR), resveratrol (RES), vitamin C (VITC), retinol (RET) and arbutin (ARB) (Sigma‐Aldrich, Germany). Loading efficiencies of RES and ARB were quantified by their fluorescence intensity as measured by the Varioskan Lux (Thermo Scientific, USA) [9].

sEVs before and after loading were fixed with 4% paraformaldehyde; scanning electron micrographs (SEM) were then obtained using the FESEM SU8010 microscope (Hitachi High‐Technologies, Japan). Immunocapture of CD81‐, CD9‐ and CD63‐positive sEVs was performed using the Leprechaun Human Tetraspanin Kit (Unchained Labs, UK) following the manufacturer's protocol. Fluorescence intensity and size of each particle were analyzed using the Leprechaun Analysis 1.2. software as previously described [9, 10].

### 2.5 UVB Irradiation With HaCaT Cells

Human keratinocyte HaCaT cells were seeded in a 96‐well plate at a density of 7 × 10^3^ cells/well for 24 h in DMEM‐10% FBS at 37°C and 5% CO_2_. The cells were then incubated with different sEV preparations or compounds for 48 h. For ROS measurement, cells were first stained with 25 μM DCFDA (ab113851, Abcam) for 45 min in the dark [9], then washed with PBS and exposed under UVB Narrowband lamps (PL‐S 9 W, Philips) at 30 mJ/cm^2^. ROS production was measured by the Varioskan LUX multimode microplate reader at 485/535 wavelength. For mRNA measurement, cells were exposed to UVB as above and rested for 24 h. Total RNA was extracted using the Quick‐RNA Microprep Kit (Zymo Research, USA), followed by cDNA synthesis using the High‐Capacity cDNA Reverse Transcription Kit (Applied Biosystems, USA). Real‐time PCR was performed by SYBR Green detection method using validated primer sequences (Table S1). HaCaT cell viability was determined using CCK8 colorimetric assay (ab228554, Abcam) [9].

### 2.6 Melanin Measurement With B16F10 Cells

Human melanocyte B16F10 cells were seeded in a 24‐well plate at a density of 1.5 × 10^4^ cells/well for 24 h in DMEM‐10% FBS at 37°C and 5% CO_2_. The cells were then incubated with different sEV preparations or compounds for 72 h. First, conditioned medium was collected to measure extracellular melanin at OD 405 nm. Cells were then lysed with 1 N NaOH solution and 10% DMSO at 80°C for 90 min, cell lysate was then collected to measure intracellular melanin at OD 405 nm. B16F10 cell viability was determined using CCK8 colorimetric assay (ab228554, Abcam) [9].

### 2.7 Statistical Analysis

For ROS measurement, the untreated HaCaT cells exposed to UVB served as the control group, their ROS level was set at 100%, and the ROS levels of all other groups were normalized to that group. For qPCR assay, the untreated HaCaT cells exposed to UVB served as the control group, their gene expression level was set at 1, and the expression fold changes of all other groups were calculated by the 2^−∆∆Ct^ method. For melanin measurement, the untreated B16F10 cells served as the control group, their melanin level was set at 100%, and the melanin levels of all other groups were normalized to that group. Unpaired student's *t*‐test was used to compare between two groups: the experimental group and the control untreated group. All statistical tests were performed using GraphPad Prism. Bar graphs indicated mean ± SEM; *p* < 0.05 was considered significant.

## 3 Results

### 3.1 Skin Anti‐Aging Effects of Human ASC‐sEVs and Natural Compounds

**FIGURE 1:**
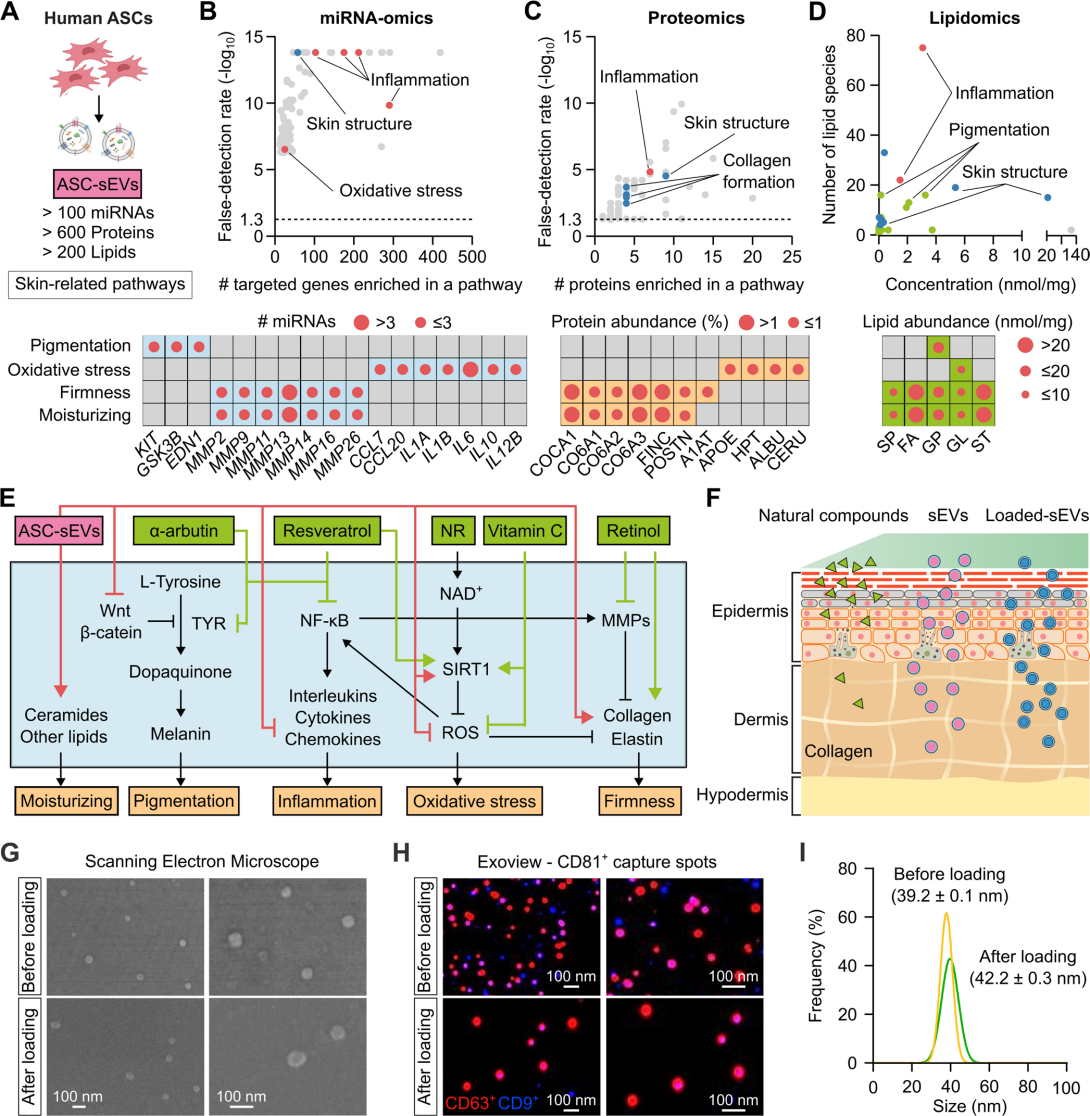
Skin anti‐aging effects of human ASC‐sEVs and natural compounds. (A) Compositions of human adipose stem cells‐derived small extracellular vesicles (ASC‐sEVs) included miRNAs, proteins and lipids that were analyzed for their functions in skin‐releated pathways. (B) miRNAs profiling and their target genes involved in skin homeostasis. (C) Proteome of human ASC‐sEVs and their functional enrichment in skin‐related pathways. (D) Lipidome and their roles in skin‐related pathways. (E) The synergistic effects of selected natural compounds and human ASC‐sEVs on molecular pathways related to skin aging. (F) Encapsulation of selected natural compounds with ASC‐sEVs could improve skin absorption and penetration. (G) Representative scanning electron micrograph (SEM) of ASC‐sEVs before and after loading with natural compounds. (H) Expression of surface markers CD63, CD81 and CD9 of ASC‐sEVs before and after compound loading. Left and right images are the same images but at different magnifications. (I) Size distribution of ASC‐sEVs before and after compound loading. MMPs, matrix metalloproteinases; ROS, reactive oxygen species; TYR, tyrosinase.

We previously developed a workflow to produce and purify ASC‐sEVs as well as generated datasets from miRNA‐sequencing, proteome and lipidome profiling of our ASC‐sEVs [9]. The ASC‐sEVs were found to contain more than 100 miRNAs, 600 proteins, and 200 lipids, which were analyzed for their biological functions related to skin health in this study (Figure 1A). For miRNAs, analysis of genes targeted by the sEV‐miRNAs showed a significant enrichment in skin structure, oxidative stress, and inflammation pathways (Figure 1B and Table S2). The top significant genes had potential functions in different aspects of skin homeostasis, such as several *MMP* genes targeted by multiple sEV‐miRNAs could affect skin firmness and moisturization. For proteins, ASC‐sEVs were enriched with proteins involved in skin structure, inflammation and collagen formation (Figure 1C and Table S3). Particularly, different types of collagen (COCA1, CO6A) and fibronectin (FINC) were abundant and could help improve skin elasticity (Figure 1C). For lipids, ASC‐sEVs were rich with diverse lipid species, particularly the fatty acids (FA) and sterol lipids (ST) (> 20 nmol/mg). Each of the lipid classes in sEVs could contribute to skin homeostasis including inflammation, skin structure, moisturization and pigmentation (Figure 1D).

Natural compounds used in cosmetic products target similar skin‐related pathways but by different mechanisms that are potentially complementary and synergistic with ASC‐sEVs (Figure 1E). For example, while sEV‐miRNAs could regulate genes involved in Wnt signaling, which in turn stablizes the master transcription factor MITF of tyrosinase TYR in melanocytes, α‐arbutin directly inhibits tyrosinase, the enzyme that converts L‐tyrosine to dopaquinone, the precursor of melanin. Furthermore, the 5 compounds NR, RES, VITC, RET and ARB were selected as they are not chemically stable or have poor cutaneous penetration. We hypothesize that encapsulating them with ASC‐sEVs could improve their skin aborption, owing to the biocompatible nature of sEVs, and synergize the biological effects of both sEVs and compound payloads (Figure 1F). Upon loading, hydrophobic compounds like RES and RET (Figure S1A) showed high loading efficiency at ~25.2% (Figure S1B), while hydrophilic compounds like ARB, NR, and VITC (Figure S1B) had a much lower loading efficiency at ~3.7% (Figure S1D). We also examined the characteristics of ASC‐sEVs before and after loading with compounds. The SEM micrograph revealed that the morphology of ASC‐sEV particles remained spherical and well‐preserved post‐loading (Figure 1G). The loaded ASC‐sEVs displayed positive surface markers CD63, CD81, and CD9, as demonstrated in the CD81 capture spots (Figure 1H). The average size of ASC‐sEVs increased to 42.2 ± 0.3 nm after compound loading, compared to 39.2 ± 0.1 nm prior to loading (Figure 1I).

### 3.2 Synergistic Anti‐Oxidant Activity of ASC‐sEVs Loaded With Natural Compounds

**FIGURE 2:**
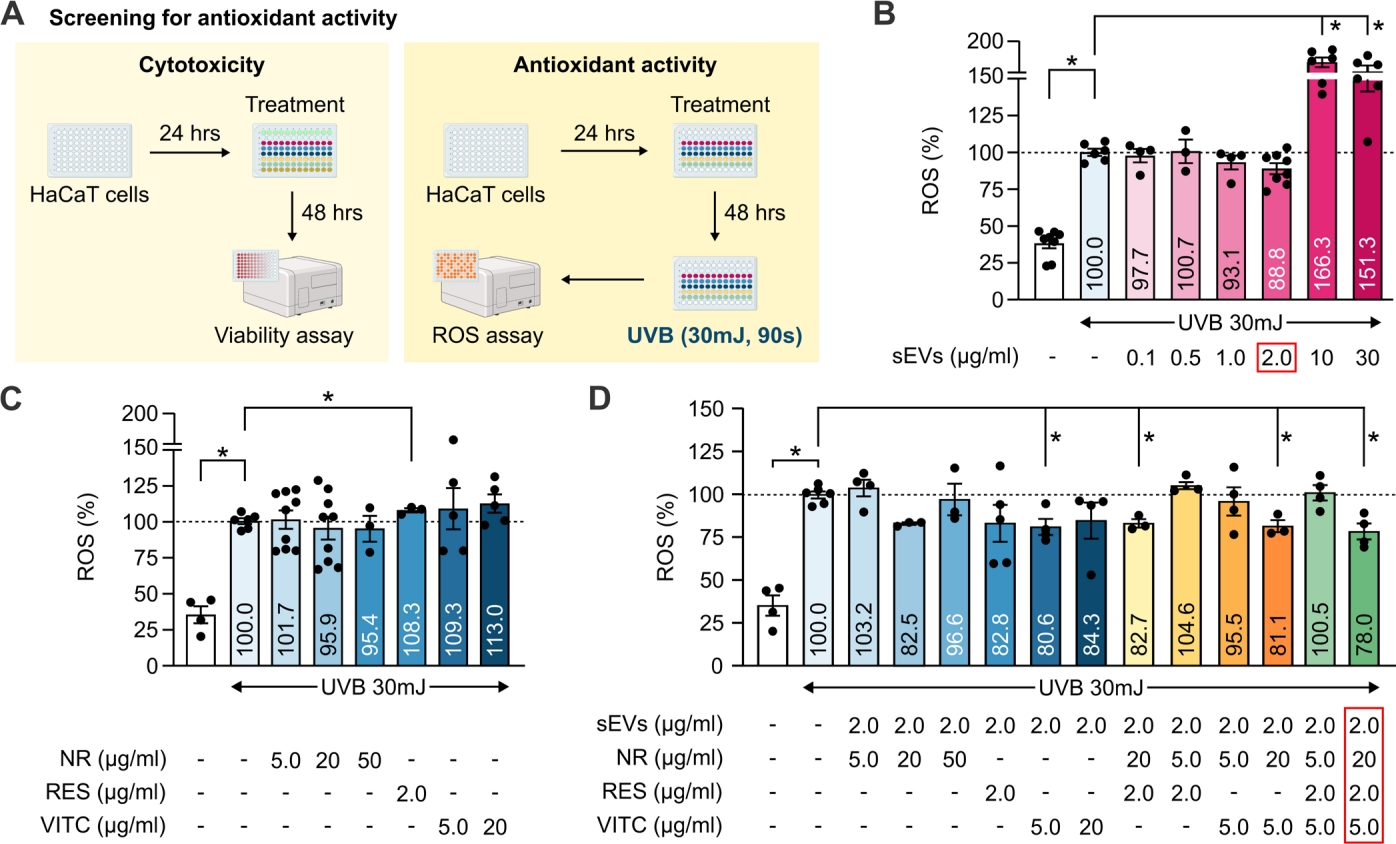
Screening for the highest combined anti‐oxidant activity. (A) Experimental design of the screen for cytotoxicity and anti‐oxidant effect using human keratinocyte HaCaT cells. ROS measurement was performed in HaCaT cells irradiated with UVB and treated with different concentrations of (B) human ASC‐sEVs, (C) selected natural compounds, and (D) human ASC‐sEVs loaded with compounds. *p < 0.05, student's *t*‐test. NR, nicotinamide riboside; RES, resveratrol; ROS, reactive oxygen species; UVB, ultraviolet B; VITC, vitamin C.

We next screened for the best combination of ASC‐sEVs and natural compounds: NR, RES and VITC for synergistic anti‐oxidant effect using the model of HaCaT cells exposed to UVB (Figure 2A). HaCaT cells were treated with either ASC‐sEVs alone, the compounds alone, or compound‐loaded ASC‐sEVs for 48 h, followed by UVB irradiation and immediate ROS measurement. The toxicity of each treatment was evaluated by HaCaT cell viability assay. Our results showed that UVB exposure led to a significant increase in ROS, and cells pre‐incubated with 2 μg/mL of sEVs showed ROS production at an average level of 88.8% compared to that of the untreated cells (Figure 2B). However, cells pre‐incubated with very high concentrations of sEVs at 10 and 30 μg/mL reversely increased ROS generation significantly more than untreated cells (Figure 2B). Therefore, we chose 2 μg/mL as the optimal concentration of sEVs for further screening. For cells pre‐treated with individual compounds, none of the treatment with NR, RES, and VitC at tested concentrations could lower ROS generation (Figure 2C). RES even induced a concentration‐dependent reduction in HaCat cell viability (Figure S2A). Combination of sEVs and natural compounds significantly decreased ROS generation in UVB‐exposed HaCat cells. The best trio‐combination was 2 μg/mL sEVs loaded with [20 μg/mL NR, 2 μg/mL RES, 5 μg/mL VITC] for 78.0% ROS production, followed by the duo‐combinations of [20 μg/mL NR, 2 μg/mL RES] and [20 μg/mL NR, 5 μg/mL VITC] for 82.7% and 81.1% ROS generation respectively (Figure 2D). Additionally, these combinations had little effects on cell viability (Figure S2B), indicating an on‐target effect on ROS reduction instead of cell viability reduction.

### 3.3 Synergistic Anti‐Wrinkle Activity of ASC‐sEVs Loaded With Natural Compounds

**FIGURE 3:**
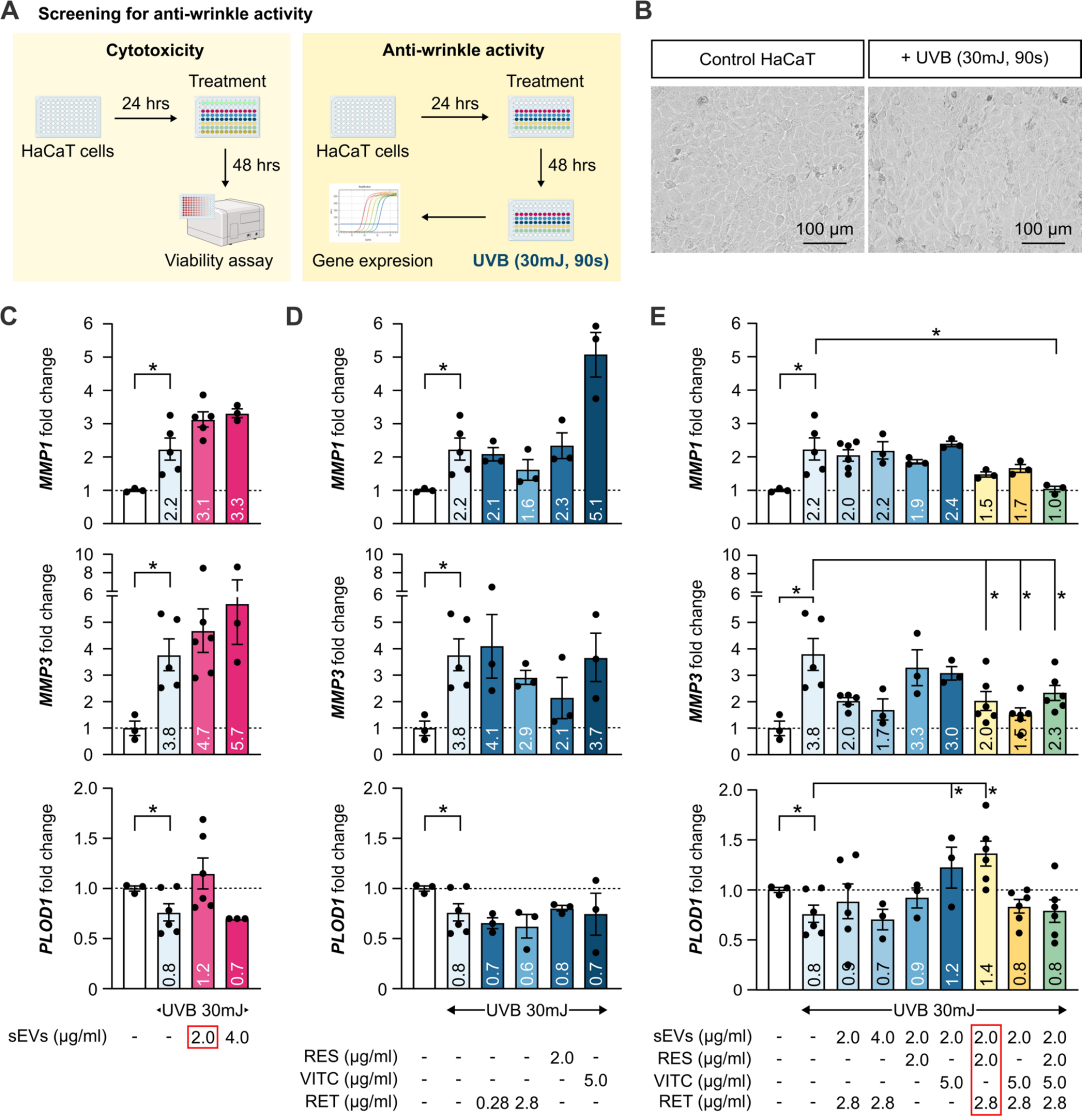
Screening for the highest combined anti‐wrinkle activity. (A) Experimental design of the screen for cytotoxicity and anti‐wrinkle effect using human keratinocyte HaCaT cells. (B) The morphology of HaCaT cells with or without UVB irradiation. Gene expressions of *MMP1*, *MMP3*, and *PLOD1* were measured in HaCaT cells irradiated with UVB and treated with different concentrations of (C) human ASC‐sEVs, (D) selected natural compounds, and (E) human ASC‐sEVs loaded with compounds. *p < 0.05, student's *t*‐test. MMP, matrix metalloproteinase; PLOD1, procollagen‐lysine 2‐oxoglutarate 5‐dioxygenase 1; RES, resveratrol; RET, retinol; UVB, ultraviolet B; VITC, vitamin C.

To evaluate the synergistic anti‐wrinkle effect of ASC‐sEVs and natural compounds: RES, VITC and RET, we treated HaCaT cels with ASC‐sEVs alone, the compounds alone, or compound‐loaded ASC‐sEVs for 48 h, followed by UVB irradiation and gene expression measurement by qPCR (Figure 3A). Upon UVB exposure and 24‐h resting, the morphology of HaCaT cells was normal with no obvious cell death (Figure 3B). We found that UVB irradiation significantly increased *MMP1* and *MMP3* while reduced *PLOD1* expression (Figure 3C). Cells pre‐incubated with sEVs or RES, VITC and RET individually did not have significant changes in *MMP1*, *MMP3*, and *PLOD1* gene expression compared to the untreated group (Figure 3C,D). When sEVs were loaded with natural compounds, the best combination of 2 μg/mL sEVs loaded with [2 μg/mL RES, 2.8 μg/mL RET] significantly reduced *MMP3* expression and upregulated *PLOD1* expression compared to the control (Figure 3E). This combination did not affect HaCat cell viability (Figure S2B).

### 3.4 Synergistic Anti‐Melanogenic Activity of ASC‐sEVs Loaded With Natural Compounds

**FIGURE 4:**
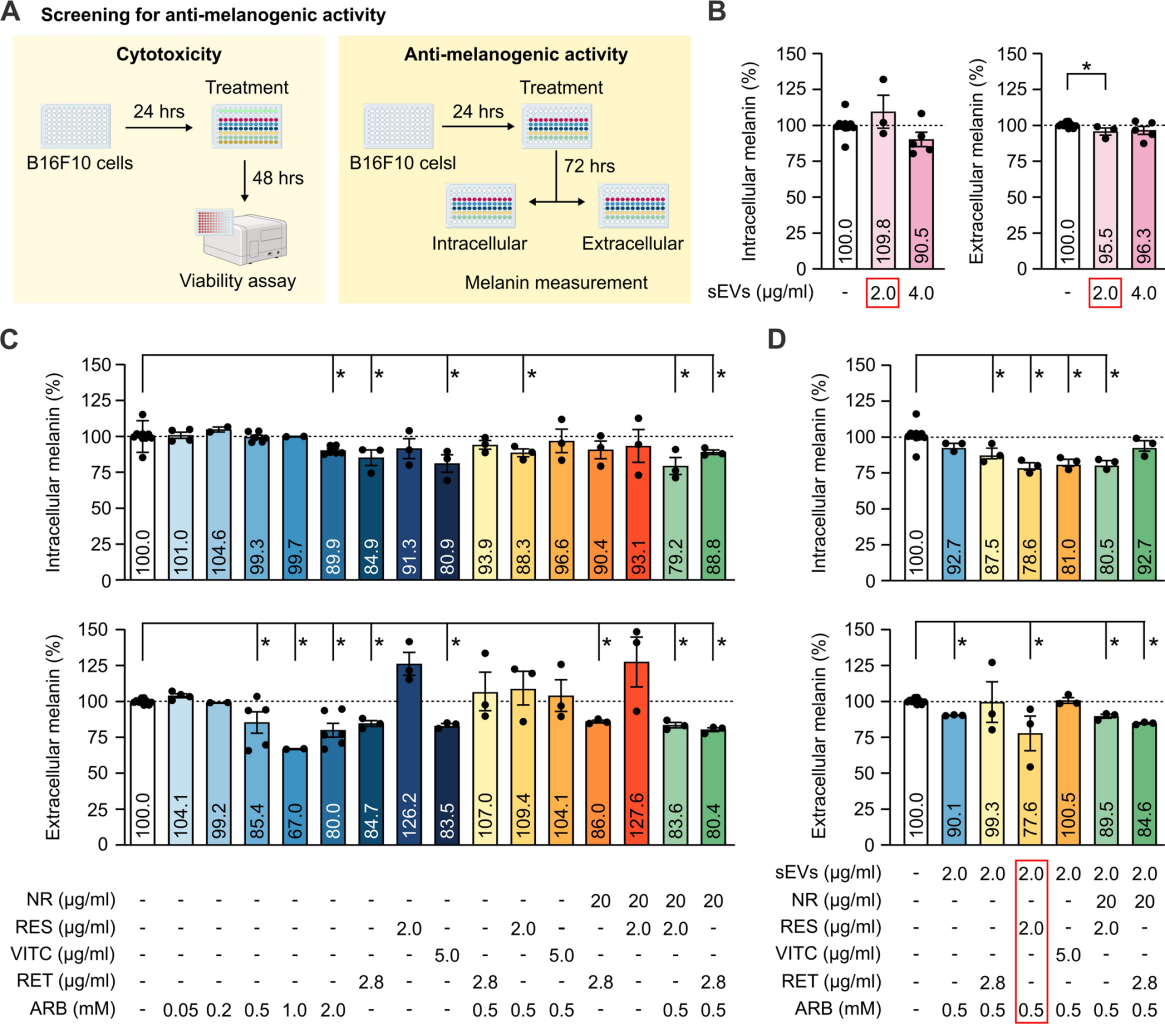
Screening for the highest combined anti‐melanogenic activity. (A) Experimental design of the screen for cytotoxicity and anti‐melanogenic effect using human melanocyte B16F10 cells. Intracellular and extracellular melanin levels were measured from B16F10 cell culture treated with different concentrations of (B) human ASC‐sEVs, (C) selected natural compounds, and (D) human ASC‐sEVs loaded with compounds. *p < 0.05, student's *t*‐test. ARB, arbutin; NR, nicotinamide riboside; RES, resveratrol; RET, retinol; UVB, ultraviolet B; VITC, vitamin C.

To investigate the anti‐melananogenic activity of ASC‐sEVs and natural compounds: RES, VITC, RET, and ARB, we measured the extracellular and intracellular melanin of the melanocytes B16F10 cells that were treated with ASC‐sEVs alone, the compounds alone, or different combinations of compound‐loaded ASC‐sEVs (Figure 4A). At the concentration of 2 μg/mL, sEVs treatment significantly reduced extracellular melanin release by 4.5% (Figure 4B), hence we chose this concentration for compound loading. Across all treatment conditions tested, several compounds and combinations significantly reduced intracellular or extracellular melanin content or both (Figure 4C). The best combination was 2 μg/mL sEVs loaded with [2 μg/mL RES, 0.5 mM ARB] that lowered intracellular and extracellular melanin content by 21.4% and 22.4% respectively (Figure 4C). This combination also did not affect B16F10 cell viability (Figure S3).

## 4 Discussion

sEVs are considered natural nanoparticles of life and gaining attentions rapidly for inherent therapeutic values in skin care and cosmetics. The biological effects of sEVs, deriving from their endogenous cargos full of heterogenous bioactive molecules, could be variable and unpredictable depending on cell sources and production workflow. We previously established a standardized protocol to produce and purify ASC‐sEVs with high batch‐to‐batch consistency and thoroughly profiled the compositions of sEV biocargos [9]. In this study, we performed pathway enrichment analysis of the endogenous miRNAs, proteins and lipids in the ASC‐sEVs, with the focus on signaling pathways regulating skin homeostasis. First, our results showed that there were no molecules directly associated with acute toxicity or skin irritation. The major sEV‐miRNAs could downregulate various matrix metalloproteinases and cytokines, which might in turn help the skin resist harmful UVB‐induced oxidative stress, inflammation and degradation of collagen matrix as previously reported [13]. The proteome of ASC‐sEVs was found rich in collagen and fibronecin, explaining the promising regenerative properties of ASC‐sEVs as already established [13, 14]. The most abundant lipid classes in ASC‐sEVs were fatty acids, sterol lipids, and ceramides, which could all be critical to maintain skin structure, elasticity and moisturization in aged skin [15, 16].

We then selected certain natural compounds that have poor solubility and stability to be encapsulated in the ASC‐sEVs to leverage the combined biological effects as well as to improve their skin absorption. While hydrophobic compounds can be loaded passively into sEVs by incubation, several studies have shown that stronger active stimulation such as sonication or electroporation was required to encapsulate hydrophilic compounds [17]. Our loading method of choise for all the compounds was sonication‐incubation as previously optimized [18]. We achieved an average loading efficiency for hydrophobic compounds of ~25%, which was in a high range compared to other studies [18, 19, 20]. As expected, hydrophilic compounds such as VITC and ARB were much more difficult to load on lipid membrane‐bound sEVs and further optimization is certainly required to improve the efficiency and cost‐effectiveness of this process. In addition, the loading efficiency for compounds that are not fluorescent in our study was extrapolated based on fluorescent compounds with similar hydrophobic/hydrophilic property; and better methods for precise quantification must be developed in the future. After exogenous compound loading, the size of sEVs slightly increased, indicating successful encapsulation or surface adsorption of the compounds, which was consistent with prior reports [21, 22].

For the in vitro screenings to identify the optimal combinations for anti‐oxidant, anti‐wrinkle and anti‐melanogenic effects, our results evidently showed that although sEVs or compounds alone both showed biological effects, the compound‐loaded ASC‐sEVs significantly outperformed the individual treatments. This suggests that sEVs might help improve the bioavailability and stability of these compounds, allowing them to exert stronger biological activities. Such effect has been reported in our previous study and also in Zhu et al. where they demonstrated enhanced anti‐aging effects of sEVs encapsulating collagen oligopeptides in skin fibroblasts [9, 23]. Moreover, it should be noted that the effective dose of the natural compounds used in our study was lower than other studies examining these compounds alone. For instance, the ROS‐neutralizing effect of RES was shown at 2.5–5.0 μg/mL [24] compared to 2.0 μg/mL in our study; and VITC was used at the concentration of 100 μM, equivalent to 17.6 μg/mL, for anti‐oxidant effect in UVB‐exposed HaCaT cells [25], compared to 5.0 μg/mL in our study. Therefore, combining natural compounds with advanced nanosystems like sEVs could be safer and more cost‐effective as lower concentrations are needed in the final formulation.

One important observation we had was the dramatic increase in ROS production when cells were treated with very high concentrations of sEVs (≥ 10 μg/mL ASC‐sEVs for 7 × 10^3^ HaCaT cells) and then exposed to UVB. Despite multiple reports for anti‐oxidant effect of ASC‐sEVs, none has reported the potential pro‐oxidant and harmful effects of sEVs when used at high doses under certain conditions. Moreover, we also found that RES reduced cell viability in a concentration‐dependent manner, aligning with one study in which RES enhanced UVB‐induced apoptosis and autophagy [26]. These findings strongly advocate for careful dose titration and optimization to maximize therapeutic benefits without inducing cytotoxicity.

In summary, our study clearly demonstrated the advantage to use sEVs to deliver both endogenous bioactive molecules and a combination of natural compounds for synergistic anti‐oxidant, anti‐wrinkle, or anti‐melanogenic effects. This approach leads to more effective and safer skincare products that achieve the desired therapeutic outcomes with minimal adverse effects. On a larger scale, it could also apply to regenerative medicine such as wound healing, where substantial scientific data corroborated that exogenously‐loaded sEVs showed improved skin regeneration and immunomodulation [3, 27]. Therefore, future studies could leverage sEVs for both their natural cargos as well as their superior capability as nanocarriers to enhance therapeutic effects.

## Author Contributions

N.V. and D.M.V. performed experiments, analyzed data and wrote the manuscript draft. N.H.B.T., D.D.N.N., P.M.P. performed data analysis and visualization. H.‐N.N. provided research resources and supervised the study. L.N.T. conceived the idea, designed the study, analyzed data and edited the manuscript. All authors have read and approved the final manuscript.

## Conflicts of Interest

LNT is a stockholder of NexCalibur Therapeutics, Corp. Other authors declare no conflicts of interest.

## Supporting information


Data S1.

## Data Availability

Data sharing is not applicable to this article as no new data were created or analyzed in this study.
