# Supplementary material for: Synergistic Anti‐Aging Effects of Adipose‐Derived Stem Cell Extracellular Vesicles Loaded With Natural Compounds
Source: J Cosmet Dermatol. 2025 Feb 9;24(2):e70021. doi: 10.1111/jocd.70021 (PMC11807792; doi:10.1111/jocd.70021)
Supplement: Supplementary file 1 — Data S1. [file JOCD-24-e70021-s001.pdf]

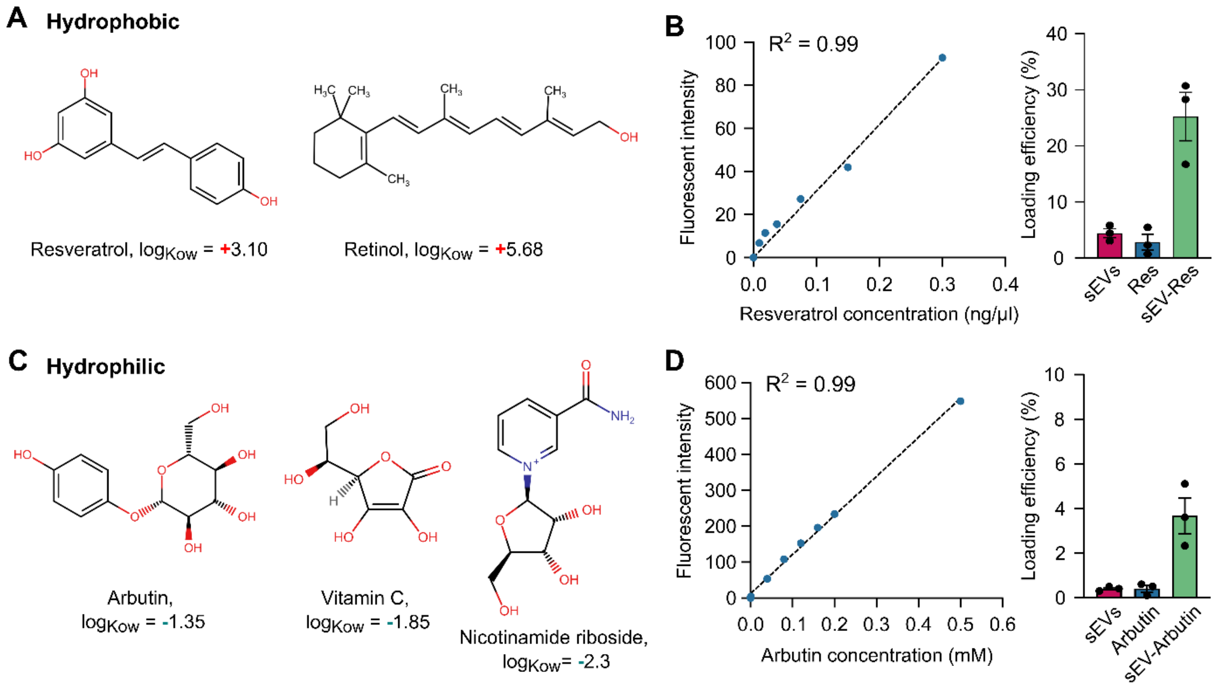

Figure S1. Natural compounds used in the study and their loading efficiency on ASC-sEVs. (A) Chemical structures and octanol-water partition coefficients (Kow) of the hydrophobic compounds: resveratrol and retinol. **(B)** The loading efficiency of representative hydrophobic compound resveratrol on ASC-sEVs was high at ~25.2%. **(C)** Chemical structures and Kow values of the hydrophilic compounds: arbutin, vitamin C, and nicotinamide riboside. **(D)** The loading efficiency of representative hydrophilic compound arbutin on ASC-sEVs was low at ~3.7%.

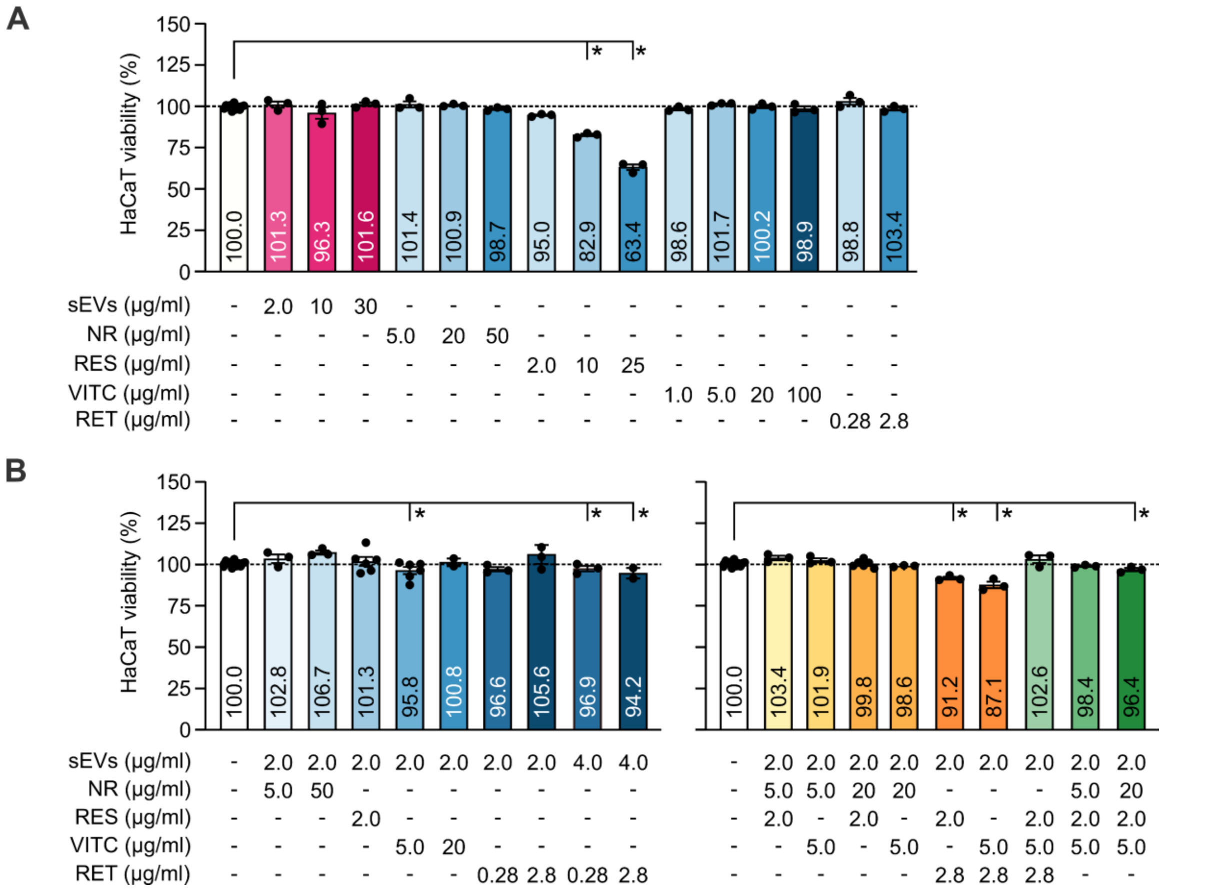

Figure S2. Cytotoxicity testing using HaCaT cells. Viability of HaCAT cells was measured after 48 hours of treatment with different concentrations of **(A)** human ASC-sEVs or selected natural compounds separately, and **(B)** human ASC-sEVs loaded with compounds. * $p < 0.05$, student's t-test. NR: nicotinamide riboside, RET: retinol, RES: resveratrol, VITC: vitamin C.

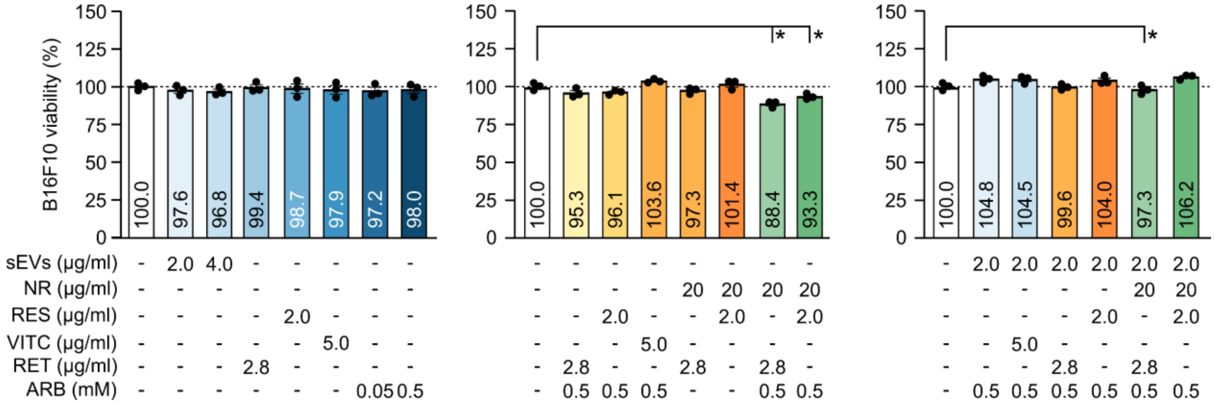

Figure S3. Cytotoxicity testing using B16F10 cells. Viability of B16F10 cells was measured after 48 hours of treatment with different concentrations of human ASC-sEVs or selected natural compounds separately, and human ASC-sEVs loaded with compounds. * $p < 0.05$, student's t-test. NR: nicotinamide riboside, RET: retinol, RES: resveratrol, VITC: vitamin C, ARB: arbutin.

Table S1. qPCR primers

Gene	Species	Strand	Sequence (5’ – 3’)
MMP1	Human	Forward	CCTAGCTACACCTTCAGTGG
		Reverse	GCCCAGTACTTATTCCCTTT
MMP3	Human	Forward	AAAGACAGGCACTTTTGGCG
		Reverse	ACGCCTGAAGGAAGAGATGG
PLOD1	Human	Forward	CGCAGACAGCTATGACGTGC
		Reverse	TCAGCAGAGAAGACCACCTG
ATCB	Human	Forward	GAGCACAGAGCCTCGCCTTT
		Reverse	TCATCATCCATGGTGAGCTGG

Table S2. Top 30 enriched pathways for genes targeted by the miRNAs in human ASC-sEVs

No.	Pathway identifier (R-HSA)	Type	Pathway name	$-\log_{10}$FDR
1	6785807	Skin-related	Interleukin-4 and Interleukin-13 signaling	13.81
2	2559583		Cellular Senescence	13.81
3	1280215		Cytokine Signaling in Immune system	13.81
4	2559580		Oxidative Stress Induced Senescence	7.51
5	2219528	Others	PI3K/AKT Signaling in Cancer	13.81
6	1257604		PIP3 activates AKT signaling	13.81
7	9006934		Signaling by Receptor Tyrosine Kinases	13.81
8	5663202		Diseases of signal transduction by growth factor receptors and second messengers	13.81
9	212436		Generic Transcription Pathway	13.81
10	73857		RNA Polymerase II Transcription	13.81
11	74160		Gene expression (Transcription)	13.81
12	449147		Signaling by Interleukins	13.81
13	5683057		MAPK family signaling cascades	13.81
14	162582		Signal Transduction	13.81
15	1266738		Developmental Biology	13.81
16	8939211		ESR-mediated signaling	12.66
17	9006925		Intracellular signaling by second messengers	12.35
18	3700989		Transcriptional Regulation by TP53	12.23
19	2428924		IGF1R signaling cascade	11.74
20	2404192		Signaling by Type 1 Insulin-like Growth Factor 1 Receptor (IGF1R)	11.61
21	199418		Negative regulation of the PI3K/AKT network	11.42
22	9006931		Signaling by Nuclear Receptors	10.81
23	5684996		MAPK1/MAPK3 signaling	10.51
24	8878159		Transcriptional regulation by RUNX3	10.25
25	9758941		Gastrulation	10.23
26	9006936		Signaling by TGFB family members	10.17
27	2219530		Constitutive Signaling by Aberrant PI3K in Cancer	10.10
28	170834		Signaling by TGF-beta Receptor Complex	10.09
29	8878166		Transcriptional regulation by RUNX2	10.08
30	2428928		IRS-related events triggered by IGF1R	10.08

Table S3. Top 30 enriched pathways for the proteins identified in human ASC-sEVs

No.	Pathway identifier (R-HSA)	Type	Pathway name	$-log_{10}FDR$
1	977606	Skin-related	Regulation of Complement cascade	4.84
2	1474244		Extracellular matrix organization	4.54
3	8948216		Collagen chain trimerization	3.70
4	2022090		Assembly of collagen fibrils and other multimeric structures	3.12
5	1650814		Collagen biosynthesis and modifying enzymes	2.93
6	1474290		Collagen formation	2.48
7	381426	Others	Regulation of Insulin-like Growth Factor (IGF) transport and uptake by Insulin-like Growth Factor Binding Proteins (IGFBPs)	9.93
8	8957275		Post-translational protein phosphorylation	9.36
9	114608		Platelet degranulation	7.04
10	76005		Response to elevated platelet cytosolic Ca2+	6.98
11	2173782		Binding and Uptake of Ligands by Scavenger Receptors	6.61
12	109582		Hemostasis	5.86
13	2168880		Scavenging of heme from plasma	5.58
14	76002		Platelet activation, signaling and aggregation	4.82
15	1474228		Degradation of the extracellular matrix	4.76
16	166658		Complement cascade	4.65
17	174577		Activation of C3 and C5	4.35
18	166663		Initial triggering of complement	4.21
19	8963898		Plasma lipoprotein assembly	4.19
20	1442490		Collagen degradation	4.19
21	3000178		ECM proteoglycans	3.93
22	216083		Integrin cell surface interactions	3.78
23	5653656		Vesicle-mediated transport	3.73
24	140877		Formation of Fibrin Clot (Clotting Cascade)	3.71
25	8963888		Chylomicron assembly	3.69
26	174824		Plasma lipoprotein assembly, remodeling, and clearance	3.54
27	8963901		Chylomicron remodeling	3.47
28	8963899		Plasma lipoprotein remodeling	3.36
29	6785807		Interleukin-4 and Interleukin-13 signaling	3.12
30	8964058		HDL remodeling	3.12
